# Potential interference of HTLV-1 HBZ protein with the DNA damage response pathway

**DOI:** 10.1186/1742-4690-8-S1-A203

**Published:** 2011-06-06

**Authors:** Torsten Wurm, Isabelle Lemasson

**Affiliations:** 1 Department of Microbiology and Immunology, East Carolina University, Greenville, NC, 27834, USA

## 

It takes decades after infection by HTLV-1 for an individual to develop adult T-cell leukemia/lymphoma. This disease is associated with increase genomic instability, due to an alteration of the DNA damage response of the infected cells. While this alteration has been attributed to the viral protein Tax, we found that the viral protein HBZ might also play a role in this process. In this study, we found that expression of HBZ in Jurkat cells increases the sensitivity of these cells to Etoposide treatment. Etoposide phosphate is an anticancer drug that induces DNA damage. This drug acts by forming a ternary complex with DNA and Topoisomerase II, leading to inhibition of the enzyme and creating double strand breaks. As a result, errors are incorporated into the DNA and the cells undergo apoptosis. We treated a stable cell line expressing HBZ, or the backbone vector, with 12.5μM Etoposide for 24 hrs. To determine the percentage of viable cells as well as cells in early or late apoptosis, we used a combination of Propidium iodide and Annexin V staining. Interestingly, we found that the cells expressing HBZ exhibit a 16% increase in total apoptotic cells and a13 % increase in late apoptotic cells compared to the cells expressing the backbone vector. This result suggests that HBZ increases DNA damage following Etoposide treatment. Current and future work investigates the effect of HBZ on other activators of the DNA damage response and tries to delineate the effect of HBZ on the regulation of the DNA damage response pathway.

